# Suction drainage in total knee replacement does not influence early functional outcomes or blood loss: a randomized control trial

**DOI:** 10.1186/s42836-022-00158-z

**Published:** 2023-02-20

**Authors:** Anton Maliarov, Nicholas Newman, Pierre Sabouret, Fidaa Al-Shakfa, Sami Chergui, Frédéric Lavoie

**Affiliations:** University of Montreal Healthcare Center (CHUM) Hospital, 1000 rue St-Denis, Montreal, QC H2X0C1 Canada

**Keywords:** drain, total knee arthroplasty, tranexamic acid, blood loss, knee range of motion

## Abstract

**Introduction:**

The use of wound drainage following total knee arthroplasty (TKA) remains controversial. The purpose of this study was to evaluate the impact of suction drainage on early postoperative outcomes in patients who underwent TKA with concomitant administration of intravenous tranexamic acid (TXA).

**Method:**

One hundred forty-six patients undergoing primary TKA with systematic intravenous TXA were prospectively selected and randomly divided into two groups. The first "Study group" (*n* = 67) received no suction drain and the second "Control" group (*n* = 79) had a suction drain. Perioperative hemoglobin levels, blood loss, complications, and length of hospital stay were assessed in both groups. Preoperative and postoperative range of motion and Knee Injury and Osteoarthritis Outcome Scores (KOOS) were also compared at a 6-week follow-up.

**Results:**

The study group was found to have higher hemoglobin levels preoperatively and during the first two days following surgery, and no difference was found between the groups on the third day. No significant discrepancies at any time were found between groups in terms of blood loss, length of hospitalization, knee range of motion, and KOOS score. Complications requiring further treatment were observed in one patient from the study group and ten patients from the control group.

**Conclusion:**

The use of suction drains after TKA with TXA did not alter early postoperative outcomes.

## Introduction

Closed suction drainage in the management of postoperative wounds after total knee arthroplasty (TKA) is considered to be a standard practice [1]. The suction time varies from a few hours, to reduce early postoperative hematomas, to a couple of days until drainage has ceased. Currently, low and high-pressure drainage systems and different techniques of clamping, regular or temporary, are available [2, 3]. Orthopedic literature has shown drains contribute to improved postoperative range of motion [4, 5], reduced soft tissue ecchymosis, alleviated intraarticular hematomas [6], better Visual Analog Scale (VAS) pain score, and diminished opioid consumption in the first postoperative days [7]. Disadvantages reportedly include increased blood loss resulting in more blood transfusions [8], higher perioperative follow-up costs, the possibility of drain dysfunction [9], more challenging rehabilitation, and prolonged hospital stay [10, 11]. The effect of drains on infection rates is still unclear. Drains are thought to have potential to prevent against infection by reducing hematoma formation and alleviating excessive pressure that can lead to hypoperfusion and delayed wound healing [12–14]. However, some believe that drains promote infectious complications through contamination via the drain port [15].

In the last decade, intravenous and topical tranexamic acid (TXA) for TKA has been shown to reduce total blood loss and the need for blood transfusion [16–18]. Intraarticular TXA was also reported to improve pain symptoms in terms of VAS score and lessen opioid use in the first 24 h following surgery [19].

The benefits of TXA raise the question of the role and necessity of closed suction drains in TKA patients. This study aimed to compare outcomes in patients with and without suction drainage following primary TKA with concomitant intravenous TXA application. We hypothesized that suction drainage would not provide any further benefit and that it would only increase the burden of care.

## Materials and methods

### Participants

This study was a prospective randomized single-center trial performed at the University of Montreal Healthcare Center (CHUM). The inclusion criteria were patients of at least 18 years who underwent primary TKA. The excluding factors were: rheumatoid arthritis, simultaneous bilateral TKA, patients refusing blood transfusions, and patients in whom TXA was contraindicated (thromboembolic syndrome, coagulopathy, or allergy). The indication for TKA was late-stage osteoarthritis with incapacitating symptoms despite conservative treatment.

Three fellowship-trained surgeons performed the surgical interventions. Recruited patients meeting the inclusion criteria underwent TKA between 2010 and 2019 after signing a consent form agreeing to participate in the study as well as adhering to a systematically-documented follow-up protocol. Patient demographics and data were recorded in a computerized database. The study (14.150) was approved by the Institutional Review Board of the hospital (CER of the CHUM, Montreal, Canada). The clinicaltrials.gov number is NCT03145493.

Patients were randomly assigned into two groups using numbered and sealed envelopes opened during the surgery. The randomization software DatInf RandList version 1.5 (Datinf GmbH, Tübingen, Deutschland) was used. The first "Study" group did not have a drain installed and the second "Control" group received postoperative suction drainage.

### Study treatments

The recruited patients underwent cemented fixed-bearing TKA with either a posterior-stabilized implant with minimal rotational constraint design (Hermes PS, Ceraver-Osteal, Roissy, France), a bicruciate-retaining prosthesis (Hermes 2C, Ceraver-Osteal, Roissy, France) or a posterior cruciate-retaining prosthesis (Triathlon, Stryker, Kalamazoo, MI, USA), depending on patient-related indications and surgeon preference. All patients received the same PVC round drain with trocar and 3-spring reservoir (10 French, 400 mL, Medline, IL, USA).

Prophylactic antibiotics were administered 15 mins before tourniquet inflation and were continued every eight hours for the first postoperative day. The pneumatic tourniquet was inflated at 300 mmHg for the surgery and was deflated after the implants were cemented in place. One gram of intravenous TXA was administered fifteen minutes before skin incision and another gram before tourniquet release. The joint was exposed via a midline skin incision. A medial parapatellar approach was used in knees with varus deformity and a lateral parapatellar approach was employed for knees with valgus deformity. The intraarticular suction drain was inserted before the closure of the joint capsule. Drains were removed on the first or second postoperative days depending on drainage.

The patients received thromboprophylaxis (unless contraindicated) in the form of either 30 mg of subcutaneous enoxaparin twice a day for 14 days, or 30 mg of subcutaneous enoxaparin every 12 h until removal of the femoral block catheter followed by rivaroxaban 10 mg daily for two weeks, according to surgeon preference. Continuous adductor canal block for twenty-four hours was performed for all patients and standard patient-controlled analgesia with opioids and NSAIDS were prescribed in both groups.

### Outcome evaluation

The primary outcome was the drop in hemoglobin levels in the first three postoperative days. The secondary outcomes were total blood loss, number of transfusions, and length of hospitalization. Complete blood counts were done preoperatively and over the first 3 postoperative days. Patients who were discharged on the second day following surgery did not undergo the third day hemoglobin evaluation. The indication for blood transfusion was hemoglobin levels less than 80 g/L or symptoms such as tachycardia, tachypnea or weakness in patients with hemoglobin levels between 80 g/L and 100 g/L.

Nadler's formula was used to estimate patients' blood volumes based on the hemoglobin balance method [20]. Height and weight were measured in meters and kilograms respectively. Estimated blood losses were calculated using Meunier's formula [21]. This formula is dependent on previously calculated blood volumes, preoperative hemoglobin (Hbi) and postoperative hemoglobin (Hbe).

$${\displaystyle \begin{array}{c}\begin{array}{l}\textbf{Estimated}\kern0.17em \textbf{blood}\kern0.17em \textbf{volume}\kern0.17em \textbf{males}\\ {}\kern3.239999em =\textbf{1000}\times \left[\left(\textbf{0.3669}\times {\boldsymbol{height}}^{\boldsymbol{3}}\right)+\left(\textbf{0.03219}\times \boldsymbol{weight}\right)+\textbf{0.6041}\right]\end{array}\\ {}\begin{array}{l}\textbf{Estimated}\kern0.17em \textbf{blood}\kern0.17em \textbf{volume}\kern0.17em \textbf{females}\\ {}\kern3.239999em =\textbf{1000}\times \left[\left(\textbf{0.3561}\times {\boldsymbol{height}}^{\boldsymbol{3}}\right)+\left(\textbf{0.03308}\times \textbf{weight}\right)+\textbf{0.1833}\right]\\ {}\;\end{array}\\ {}\boldsymbol{Estimated}\kern0.17em \boldsymbol{blood}\kern0.17em \boldsymbol{loss}=\boldsymbol{Blood}\kern0.17em \boldsymbol{volume}\times \frac{{\boldsymbol{Hb}}_{\boldsymbol{i}}\hbox{-} {\boldsymbol{Hb}}_{\boldsymbol{e}}}{{\boldsymbol{Hb}}_{\boldsymbol{e}}}\end{array}}$$

Passive knee flexion and extension were recorded and compared between groups preoperatively, immediately following surgery, on the third postoperative day, and at the six-week follow-up. The standard goniometer method was used to determine the degree of motion. The physiotherapy protocol with active and passive exercises was administrated from the first postoperative day.

The KOOS score was used to subjectively evaluate knee function before surgery and at the six-week follow-up. The KOOS questionnaire is a condition-specific patient-reported outcome developed to investigate the patients' burden due to knee complaints. The KOOS includes five subscales measuring different knee-specific domains: pain, symptoms, activities of daily living (ADL), sports and recreational activity, and quality of life (QOL) [22].

### Statistical analysis

The data were recorded and summarized using Microsoft Excel 2010 (Microsoft Corp., Redmond, WA, USA). Statistical analysis was performed using SPSS software (version 25, IBM Corporation). The student *t*-test was used to compare continuous variables from both groups and significance was set at a *P*-value less than 0.05. The assumptions were made based on random groups, adequacy of sample size, and equality of variance in standard deviation. A minimum sample size of 140 patients according to a power study aimed at detecting a difference in hemoglobin level of 5 mg/L, considering an alpha value of 0.05, a beta value of 0.2, a standard deviation of 10 mg/L, and a dropout rate of 10%. The chi-square test was used to compare categorical data between the groups. Means and standard deviations were calculated for normally-distributed variables and frequencies were measured for categorical variables.

## Results

**Fig. 1:**
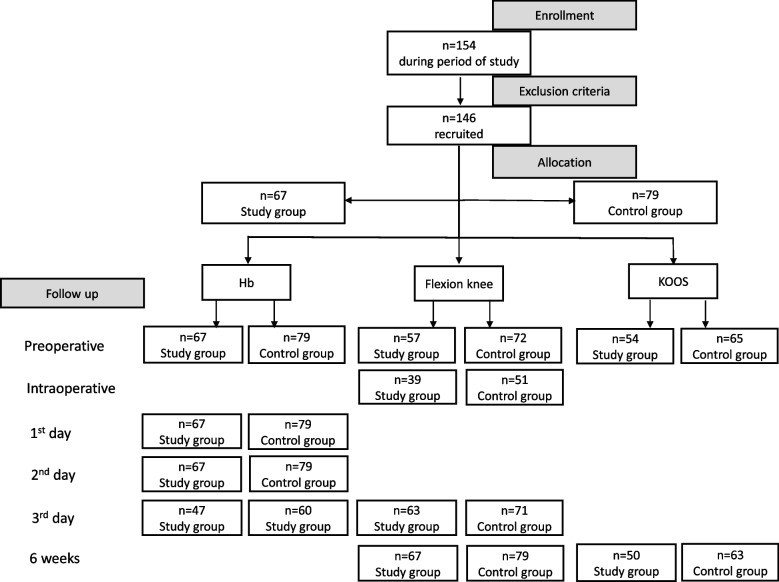
Flowchart divided by periods of study. (KOOS = Knee injury and Osteoarthritis Outcome Score; Hb = Hemoglobin level)

A total of 146 knee replacements were performed in 146 patients (46 males and 100 females) during the study period. The study group was composed of 67 patients and the control group was formed of 79 patients (Fig. 1). The mean age of the cohort was 69 years (average 52–84 years). No significant difference in gender, age, body mass index (BMI), or types of surgery was noted between the groups.

### Hemoglobin drop evaluation

Hemoglobin levels were measured in all patients preoperatively and on the first and second days after surgery. In 107 patients, their hemoglobin was measured on the third day (Table 1). The study group was shown to have higher hemoglobin levels preoperatively at a mean of 138.6 ± 10.8 g/L compared to controls with a mean hemoglobin of 134.1 ± 12.3 g/L (*P* = 0.023). The study group had a mean hemoglobin of 116.7 ± 12.6 g/L and 109.9 ± 12.7 g/L on the first and second postoperative days respectively. Patients in the control group were shown to have lower postoperative hemoglobin measurements, with a mean of 112.4 ± 12.5 g/L, followed by 104.3 ± 16.8 g/L for the first two days following surgery (*P* = 0.045, *P* = 0.027). On the third day no difference was found in hemoglobin levels between the two groups (*P* = 0.083).

**Table 1:** Mean hemoglobin level (Hb) and estimated blood loss evaluation divided between the two study groups

Variable	Groups	Number of patients	Mean ± Standard deviation	*P* value
Hb preoperatively (g/L) ^*a*^	Study	67	138.6 ± 10.8	0.023
	Control	79	134.1 ± 12.3	
Hb 1^st^ day (g/L)^*a*^	Study	67	116.7 ± 12.6	0.045
	Control	79	112.4 ± 12.5	
Hb 2^nd^ day (g/L)^*a*^	Study	67	109.9 ± 12.7	0.027
	Control	79	104.3 ± 16.8	
Hb 3^rd^ day (g/L)	Study	47	108.2 ± 14.7	0.083
	Control	60	103.5 ± 12.6	
var Hb 1	Study	67	-21.9 ± 7.8	0.876
	Control	77	-21.7 ± 7.6	
var Hb 2	Study	67	-28.6 ± 8.8	0.627
	Control	77	-29.8 ± 17.7	
var Hb 3	Study	67	-65.9 ± 51.9	0.147
	Control	76	-53.8 ± 45.8	
Estimated blood volume (mL)	Study	61	5242.2 ± 1250.0	0.178
	Control	63	4928.9 ± 1321.2	
Estimated blood loss (mL) 2^nd^ day	Study	61	1363.9 ± 567.1	0.774
	Control	60	1332.1 ± 647.7	
Estimated blood loss (mL) 3^rd^ day	Study	40	1421.1 ± 577.1	0.965
	Control	48	1414.3 ± 807.8	
No of patients transfused	Study	0		
	Control	1		
No of pack cell transfused (units)	Study	0		
	Control	1		

Data are shown as means ± standard deviation or numbers.

^*a*^ Statistically significant difference between groups

### Blood loss evaluation

The estimated total blood loss on the second day after TKA in the study group and the control group was 1363.9 ± 567.1 mL and 1332.1 ± 647.7 mL, respectively (*P* = 0.774). On the third postoperative day, the study group had a mean total blood loss of 1421.1±577.1 mL and the control group had a mean loss of 1414.3 ± 807.8 mL (*P* = 0.965). Thus, no statistically significant differences in blood loss were observed between groups. One 84-year-old patient with the installation of a drain, who had a cardiac history, received a blood transfusion. The drainage volume was 110 mL, which was not greater than from other drained knees.

### Knee range of motion

Preoperative evaluation revealed no differences between groups in the range of motion (*P* = 0.911). In all postoperative follow-ups, patients with and without drains demonstrated similar ranges of flexion. Knee flexion in all patients improved between discharge and the six-week clinical visit. However, mean ranges of flexion in both groups did not return to preoperative baseline. The control group was observed to have greater hyperextension upon examination immediately following surgery (-0.4 *vs.* 0.5*)* (*P =* 0.030) (Table 2).

**Table 2:** Mean knee range of motion (degrees) divided between two groups

	Groups	Number of patients	Mean ± Standard deviation	*P*-value
Preoperative flexion (°)	Study	57	124.7 ± 15.0	0.911
	Control	72	124.4 ± 14.5	
Preoperative loss of extension (°)	Study	49	3.3 ± 5.4	0.107
	Control	60	1.8 ± 4.1	
End of the surgery flexion (°)	Study	39	137.1 ± 9.8	0.572
	Control	51	136.0 ± 7.2	
End of the surgery loss of extension (°)^*a*^	Study	39	0.5 ± 2.2	0.030
	Control	51	-0.4 ± 2.0	
Flexion on the 3^rd^ day (°)	Study	63	87.4 ± 20.4	0.996
	Control	73	87.5 ± 17.1	
Loss of extension on the 3^rd^ day (°)	Study	63	1.0 ± 12.1	0.613
	Control	73	0.05 ± 11.0	
Flexion on 6 week follow-up (°)	Study	67	110.1 ± 13.5	0.073
	Control	79	105.4 ± 17.3	
Loss of extension on 6 week follow-up (°)	Study	47	3.0 ± 3.8	0.220
	Control	60	2.0 ± 4.9	

Data are shown as mean ± standard deviation or numbers.

^*a*^ Shows a statistically significant difference between groups

### KOOS Scores

**Fig. 2:**
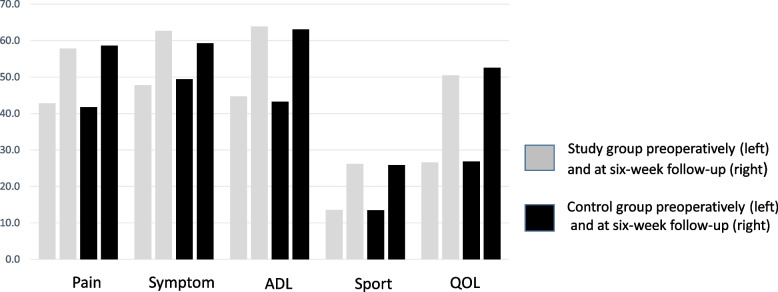
Preoperative and six-week follow-up KOOS of the two groups. (KOOS = Knee injury and Osteoarthritis Outcome Score; ADL = Activities of Daily Living; QOL= Quality of Life)

119 questionnaires from preoperative time and 113 questionnaires from the six-week follow-up were available for evaluation. Mean KOOS scores improved in all subscales without significant differences between groups (Table 3). Preoperative and postoperative KOOS scores were plotted for both groups **(**Fig. 2).

**Table 3:** Knee injury and Osteoarthritis Outcome Scores (KOOS) of the two groups preoperatively and at six-week follow-up

	Pain	Symptom	ADL	Sport	QOL
Preoperative KOOS					
Study group (54 pts)	42.7 ± 16.6	47.7 ± 17.4	44.7 ± 18.1	13.5 ± 14.6	26.5 ***± ***18.2
Control group (65 pts)	41.7 ± 17.3	49.4 ± 18.5	43.1 ± 18.9	13.4 ± 16.6	26.8 ***± ***20.2
*P* value	0.736	0.627	0.654	0.975	0.935
**KOOS at six- week follow-up**					
Study group (50 pts)	57.7 ± 15.9	62.6 ± 14.9	63.9 ± 15.9	26.1 ± 27.0	50.4 ***± ***22.9
Control group (63 pts)	58.6 ± 19.8	59.3 ± 16.5	63.0 ± 19.2	25.8 ± 23.0	52.5 ***± ***26.0
*P* value	0.807	0.265	0.805	0.965	0.656

Data are shown as mean ± standard deviation.

*ADL* activities of daily living, *QOL* quality of life

### Length of hospitalization

The mean length of hospital stay in the study group was 3.1 days and it was 3.5 days in the control group. No difference was observed between groups (*P* = 0.352). A total of 39 patients, 21 in the study group and 18 in the control group, had a two-day hospital stay (*P* = 0.265).

### Complications

Eleven patients (7.5%) developed a complication that required further intervention. One patient was from the study group and ten patients were from the control group (*P* = 0.010). Three knees in a drained group showed early signs of infection that were treated with incision and drainage, polyethylene exchange, and intravenous antibiotics for a minimum of six weeks. Three knees in a drained group demonstrated wound inflammation and received oral antibiotic treatment. The remaining five patients (one from the study and four from the control group) presented at the six-week follow-up with a stiff knee and were treated with manipulation of the knee or arthroscopic release with manipulation.

## Results

The main purpose of this study was to compare the evolution of hemoglobin levels among patients with and without suction drainage following primary TKA using intravenous TXA. This study showed a higher hemoglobin level preoperatively in the group of patients without drain installation. Hemoglobin levels normalized between groups on the third postoperative day and no significant differences in terms of estimated blood loss were observed following TKA. Perioperative blood losses after TKA included intraoperative bleeding and postoperative drainage. Additionally, hidden blood losses are defined as extravasation into the surrounding soft tissue, hemolysis, and residual blood in the joint space, which may account for half of the total blood loss [23].

Similarly, Rajesh *et al.* conducted a randomized control trial (RCT) in TKA patients with concomitant TXA application. They observed no significant effect of suction drainage on perioperative bleeding [7]. Conversely, an RCT by Wang *et al.*, which followed 80 patients with TKA and TXA, highlighted that hidden blood losses were significantly greater in the group without suction drainage [10]. In contrast, Chen*et al.* demonstrated, in 1660 patients without TXA, that drainage was associated with more perioperative total blood loss following TKA. However, these additional blood losses did not translate into an increased transfusion rate [24]. The findings of Chen *et al.* might be explained by the negative pressure induced by suction drainage which could detach hemostatic clots and aggravate bleeding.

Despite reports of drains leading to increased postoperative bleeding, the use of drains in TKA patients has been proposed to diminish the risk of hematoma. In the absence of a drain, hematoma formation can occur during the first few hours postoperatively, possibly leading to the stretching of the knee capsule and pain from the surrounding structures. Additionally, hematoma formation may affect rehabilitation programs with decreased range of motion in the early postoperative period and lead to a persistent loss in knee range of motion [25].

Tranexamic acid is an analog of the amino acid lysine, which can competitively inhibit plasminogen activation and plasmin binding to fibrin, thus inhibiting fibrinolysis. The contribution of TXA to reducing bleeding and consequently diminishing hematoma formation leads to reduced analgesic drug consumption and fewer blood transfusions after surgery [19].

The effects of drains on postoperative knee function are still debated. Andrade *et al*. observed that drain usage led to better functional outcomes in 42 patients who also received TXA [ 5]. On the other hand, a previous study by Wang *et al.* reported better early functional outcomes without draining [10]. Watanabe *et al*. did not see any contributions from suction drainage to postoperative ranges of motion in 63 patients with TXA administration [26]. Moreover, Yin *et al.* followed 111 patients without TXA and similarly reported that drainage did not lead to any differences in terms of the postoperative range of motion [27]. In the current study, all patients had knee flexion improvement at the six-week follow-up when compared to flexion at discharge. There was no significant difference in the range of flexion between groups in all evaluations. However, a higher incidence of stiff knees requiring manipulation was noted in the drain group at the six-week follow-up*.* The greater hyperextension seen at the end of surgery in patients with drains is not thought to have a clinical impact*.* Differences in hyperextension between groups became negligible at the six-week follow-up.

The impact of suction drainage on the length of hospitalization following TKA is still unclear. Nishitani *et al*. conducted a study with 166 knees and noticed that drain usage, age, and comorbidities were related to the length of hospital stay [11]. The RCT performed by Wang *et al*. with 80 knees demonstrated shorter hospital stays in the non-drain group [10]. In contrast, Chen*et al.*, who conducted a retrospective study encapsulating 1660 patients, showed no difference in both groups of patients in the duration of hospitalization [24]. In our study, intraarticular drainage did not affect the length of hospitalization.

In a study by Demirkale *et al.*, the infection rate was found to be lower in patients without drains [28]. Andrade *et al.* evaluated 42 patients and noticed similar incidences of infection as three patients in drain group and one in the non-drain group developed infections [5]. In addition, Li *et al.* observed no difference between drain and non-drain groups in terms of infection rates when following 100 patients [14]. Rajesh *et al*. reported that none of the 35 recruited patients developed a surgical site infection [7]. The current study reported three patients from the drain group with early signs of knee infection that required additional surgery compared to none in the treatment group, suggesting a disadvantage to suction drainage.

The current study had some limitations. First, a third of the patients were discharged on the second day, so the third-day hemoglobin levels were not available for all patients. However, based on the hemoglobin levels of the first two postoperative days, it appears unlikely that a significant difference would have been found even with this missing data. Second, there was heterogeneity in the cohort regarding surgical technique and implant types because three different surgeons operated on patients. Nonetheless, as this heterogeneity applied equally to both study groups because of randomization, it probably did not affect the study findings.

## Conclusion

The application of intraarticular suction drainage following TKA with TXA use did not show any effects on postoperative hemoglobin levels, blood loss, prosthetic knee range of motion, KOOS score, and the length of hospital stay. However, we observed that drains were associated with a greater complication rate: infection and knee stiffness. The usage of a suction drain may not provide any benefit and may have adverse effects.

## Data Availability

The data and related materials are available and stored in the CHUM, Montreal, Canada. The full address is CHUM hospital, 1000 rue St-Denis, Montreal, Quebec, H2X0C1, Canada.
